# Efficacy of traditional Chinese massage therapy combined with Schroth therapy in patients with adolescent idiopathic scoliosis: a meta-analysis

**DOI:** 10.3389/fped.2026.1638835

**Published:** 2026-04-17

**Authors:** Boyun Huang, Yunrui Jin, Rongping Ye, Yuhui Wang, Shining Peng, Qian Tang, Shanzhi Ma

**Affiliations:** 1 Sports and Joint Orthopedics Department, Fuzhou Hospital of Traditional Chinese Medicine, Fuzhou, China; 2 Department of Rehabilitation, Chongqing Orthopedic Hospital of Traditional Chinese Medicine, Chongqing, China; 3 School of Acupuncture Moxibustion and Tuina, Tianjin University of Traditional Chinese Medicine, Tianjin, China; 4 School of Integrative Medicine, Tianjin University of Traditional Chinese Medicine, Tianjin, China

**Keywords:** adolescent idiopathic scoliosis, massage therapy, meta-analysis, Schroth therapy, systematic review

## Abstract

**Background:**

Adolescent idiopathic scoliosis (AIS) is a three-dimensional structural deformity of the spine that arises due to genetic factors and unhealthy lifestyle habits during the adolescent growth period, prior to skeletal maturity. This condition significantly affects both the physical development and mental well-being of adolescents. Both traditional Chinese massage therapy and schroth therapy have been clinically validated as effective interventions for scoliosis. However, whether the combination of these two approaches yields superior therapeutic outcomes remains controversial. Therefore, this study employed a meta-analysis to evaluate the efficacy of traditional Chinese massage therapy combined with Schroth therapy in the treatment of AIS.

**Methods:**

We systematically searched multiple databases, including the Cochrane library, Web of Science, Embase, PubMed, CNKI, and Wanfang database, for randomized controlled trials (RCTs) investigating the effects of traditional Chinese massage therapy combined with Schroth therapy on AIS, with the search period extending up to January 2025. Two reviewers independently screened the literature, extracted relevant data, and assessed the methodological quality of the included studies. Statistical analyses were performed using Stata 14.0 to conduct the meta-analysis. The Grading of Recommendations Assessment, Development and Evaluation (GRADE) system was utilized to assess the quality of evidence for each outcome.

**Results:**

A total of eight studies involving 549 patients were included. The meta-analysis revealed that the combination of traditional Chinese massage and schroth therapy significantly improved overall treatment outcomes for AIS [RR = 1.16, 95%CI(1.01, 1.32), *P* = 0.0146; Level of evidence: very low], enhanced scoliosis research society−22 questionnaire scores [SMD = 2.44, 95%CI(0.68, 4.20), *P* = 0.007; Level of evidence: very low] and reduced the Cobb Angle [SMD = −1.23, 95%CI(−2.10, −0.35), *P* = 0.006; Level of evidence: very low].

**Conclusion:**

Current evidence suggests that traditional Chinese massage therapy combined with Schroth therapy may offer greater clinical benefits than Schroth therapy alone for patients with AIS. However, owing to the limited number and methodological quality of the included studies, these findings should be interpreted with caution and further validated in high-quality research.

## Introduction

Adolescent idiopathic scoliosis (AIS) is one of the most common structural spinal deformities affecting adolescents. It is a progressive condition, accounting for 84%–89% of all scoliosis cases, with a higher incidence and greater severity observed in girls (1, 2). During adolescence, the spinal column undergoes rapid growth, while the early clinical manifestations of AIS often remain inconspicuous. As a result, the condition is frequently diagnosed only after significant progression has occurred, which not only compromises the physical appearance and posture of affected individuals but also adversely impacts their overall growth and development (3). The clinical consequences of AIS are characterized by insidious onset, progressive deterioration, and lifelong implications (4, 5). As the Cobb angle increases, patients commonly develop postural asymmetries, including uneven shoulders, prominent scapulae, thoracic asymmetry, and rib humps (6). These visible alterations not only undermine patients' self-esteem but may also precipitate psychological distress such as anxiety and social phobia, thereby severely diminishing quality of life (7). In cases of severe curvature, particularly in thoracic scoliosis, the distorted thoracic cage can restrict pulmonary and cardiac function. Over time, this may lead to reduced lung capacity, dyspnea, and impaired circulatory efficiency (6). In extremely advanced stages, spinal deformity may result in spinal cord compromise or irreversible neurological damage, leading to permanent functional impairment (8–10). Thus, AIS represents more than a mere skeletal abnormality; it exerts profound effects on adolescents' physical health, psychological well-being, social adaptation, and family economic status. The etiology of AIS remains incompletely understood, and is likely multifactorial, involving genetic predisposition, endocrine dysregulation, biomechanical factors, neurological abnormalities, and muscular imbalances, among others (11–14). Currently, AIS has emerged as a major public health concern affecting adolescent physical health, underscoring the critical importance of early detection, accurate diagnosis, and timely intervention.

Clinical management strategies for scoliosis are broadly categorized into surgical and conservative approaches, with the choice of treatment guided by patient age, magnitude of the curvature, and risk of progression (15). For patients with a Cobb angle of less than 45°, non-surgical intervention is clinically recommended and widely acknowledged for its efficacy in improving health-related quality of life in individuals with mild to moderate scoliosis, as well as in halting further curve progression (16, 17). Conservative treatment modalities include physiotherapeutic scoliosis-specific exercises (PSSE), bracing, massage therapy, traction therapy, electrical stimulation, acupotomy, acupuncture, and other rehabilitative techniques (18–20). A retrospective study demonstrated that chiropractic intervention could reduce the Cobb angle in patients with mild to moderate AIS (20). PSSE primarily constitutes a behavioral approach centered on active self-correction, spinal stabilization, and patient education (21). It emphasizes three-dimensional postural correction, training for spinal stability, and the integration of corrected postures into daily activities (22). Although bracing and PSSE constitute the cornerstone of conservative management for AIS, challenges such as the high cost of braces and difficulties in maintaining long-term adherence to exercise regimens persist (23–25). In clinical practice, to retard curvature progression and achieve more comprehensive therapeutic outcomes, PSSE is often combined with passive therapies or integrated with various forms of physical training, such as traditional health exercises, bracing, massage, and physical agent modalities (26). Among the various PSSE approaches, Schroth therapy is one of the most extensively studied and widely utilized exercise-based interventions (2, 21, 27). This method specifically targets the three-dimensional rotational deformity characteristic of scoliosis and formulates individualized treatment plans tailored to each patient's unique curvature pattern (28, 29). Schroth therapy achieves correction of abnormal spinal curves through a combination of stretching exercises that oppose the direction of curvature, along with targeted muscle activation and specific breathing techniques (21, 28). Evidence indicates that Schroth therapy is effective in reducing Cobb angles and enhancing quality of life in patients with AIS (30).

Traditional Chinese medicine has long recognized AIS, and traditional Chinese massage, as a relatively safe therapeutic modality, occupies an important role in the conservative management of AIS by effectively reducing Cobb angles and preventing curve progression (31). Relevant studies have shown that for AIS patients presenting with cervical dystonia, chiropractic spinal manipulation can improve spinal alignment, alleviate pain, and enhance quality of life (32). A meta-analysis conducted by Li et al. demonstrated that massage therapy yields significant benefits in improving both structural and clinical outcomes in AIS (33).

In clinical settings, both traditional Chinese massage and Schroth therapy have been validated as effective interventions for AIS through multiple studies (34–37). However, it remains uncertain whether combining these two therapies can produce synergistic effects that lead to substantially improved treatment outcomes. The efficacy of combined traditional Chinese massage and Schroth therapy for AIS is still debated, and no consensus has been reached. Therefore, the present study aims to evaluate the therapeutic efficacy of traditional Chinese massage combined with Schroth therapy in the management of AIS through a meta-analytic approach, thereby providing a foundation for future clinical research in this area.

## Materials and methods

This meta-analysis was conducted in accordance with the Preferred Reporting Items for Systematic Reviews and Meta-Analyses (PRISMA) 2020 statement (38, 39).

### Search strategies

1. Database source: The Cochrane library, Web of Science, Embase, PubMed, CNKI and Wanfang database;
2. Literature content: Randomized Controlled Trials (RCTs) investigating traditional Chinese massage therapy combined with schroth therapy for AIS;
3. Search methods: Two researchers independently performed the literature search using a combination of Medical Subject Headings (MeSH) terms and keywords;
4. Search period: Databases were searched from their inception up to January 2025;
5. Search terms: massage, massage therapy, schroth, tuina, adolescent, scoliosis, idiopathic scoliosis, random*,etc.

### Inclusion criteria

Studies were included based on the following PICOS criteria:

Population (P): Adolescents diagnosed with idiopathic scoliosis.

Intervention (I): Traditional Chinese massage therapy combined with Schroth therapy.

Comparator (C): Control group receiving either traditional Chinese massage therapy alone or Schroth therapy alone.

Outcomes (O): ① Cobb angle; ② Scoliosis Research Society-22 (SRS-22) score; ③ Total effective rate.

Study design (S): RCTs.

### Exclusion criteria

Studies were excluded if they met any of the following criteria:

1. Duplicate publications identified across databases.
2. Interventions combined with other therapies not specified in the inclusion criteria.
3. Reviews, case reports, conference abstracts, or studies lacking original data.

### Study selection and data extraction

Two researchers independently screened the literature and extracted relevant materials and data. Retrieved records were imported into EndNote for duplicate removal. Initial screening was performed based on titles and abstracts, followed by full-text review according to the predefined inclusion and exclusion criteria. Any disagreements between the two researchers were resolved through discussion or consultation with a third researcher. Extracted data included: title, first author, publication year, intervention details, sample size, and outcome measures.

### Risk of bias in individual studies

The Cochrane Risk of Bias tool was employed to evaluate methodological quality of the included trials across the following domains: random sequence generation, allocation concealment, blinding of participants and personnel, blinding of outcome assessment, completeness of outcome data, selective reporting, and other potential sources of bias. Each domain was categorized as low risk, high risk, or unclear risk of bias.

### Data analysis

Statistical analyses were performed using Stata 14.0 software. For dichotomous outcomes, relative risk (RR) with 95% confidence intervals (CI) was calculated. Continuous outcomes were expressed as standardized mean differences (SMD) with 95% CI. Heterogeneity across studies was assessed using the *I*^2^ statistic. When *P* > 0.1 and *I*^2^ < 50%, a fixed-effects model was applied; otherwise, a random-effects model was employed (40). Subgroup analyses were performed according to intervention duration (≥6 months vs. <6 months). Sensitivity analyses were performed to evaluate the robustness of the pooled results. Publication bias was assessed using Egger's test and Begg's test.

### Evidence quality assessment

The Grading of Recommendations Assessment, Development, and Evaluation (GRADE) system was utilized to evaluate the quality of evidence for each pooled outcome. Evidence was assessed based on five domains: risk of bias, indirectness, inconsistency, imprecision, and publication bias. The quality of evidence was classified into 4 levels: very low, low, moderate and high.

## Results

### Literature selection and characteristics of included studies

**Figure 1:**
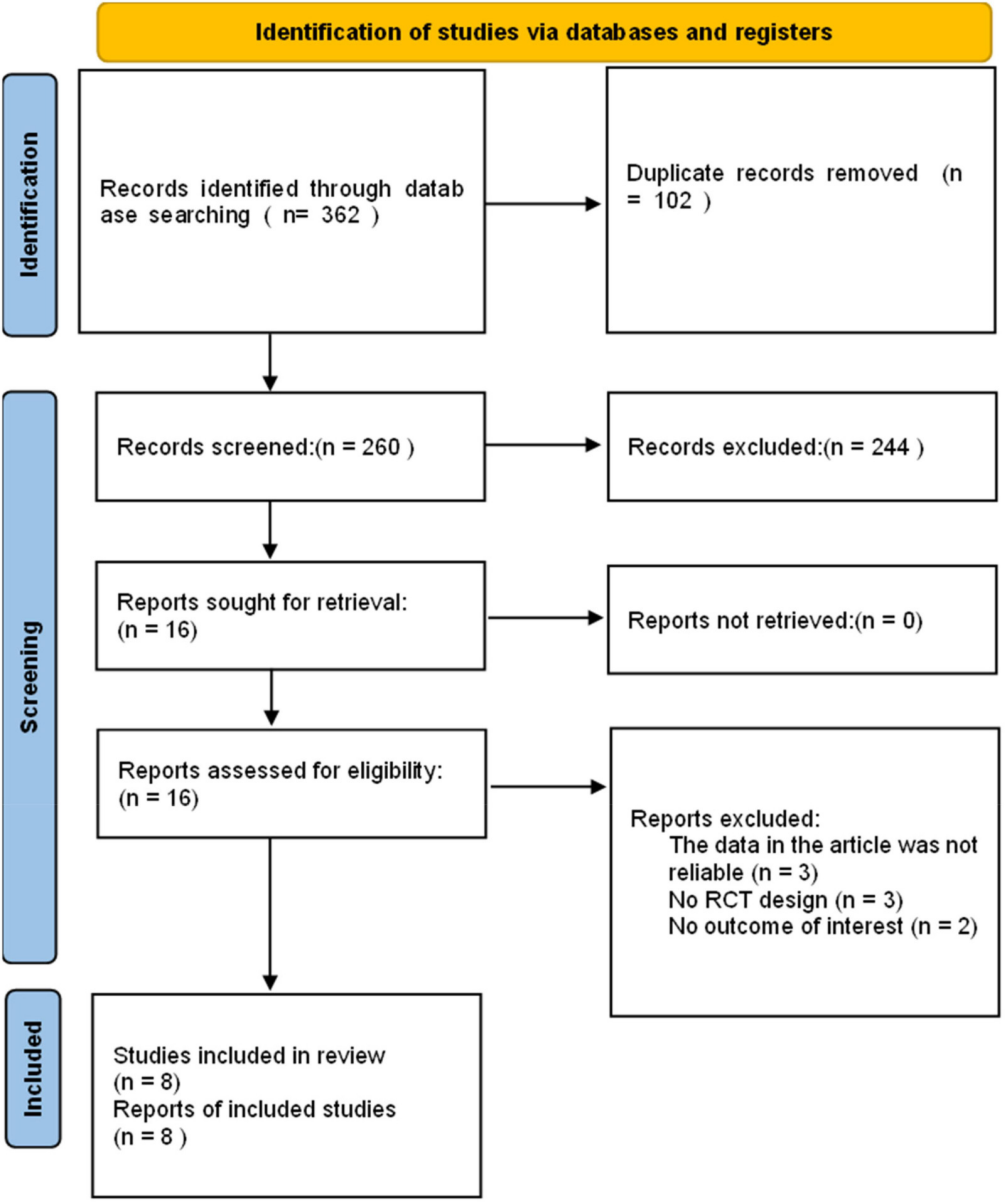
Flow diagram of the study selection process for the meta-analysis.

A total of 362 relevant records were identified through database searching. After removing 102 duplicates, 260 records remained for title and abstract screening, leading to the exclusion of 244 irrelevant studies. Following full-text review of the remaining 16 articles, 8 studies were excluded based on the inclusion and exclusion criteria. Ultimately, 8 studies (23–25, 34–37, 41) involving 549 patients were included in the meta-analysis (Figure 1). The basic characteristics of the included studies are summarized in Table 1.

**Table 1:** Basic features of included studies.

Inclusion study	Duration	Sample size(n)			Age(Years)		Intervention duration	Interventions	
		Treatment group	Control group	Treatment group	Control group	Treatment group		Control group
Pan et al. (2024) (34)	From January 2020 to January 2023	49	49	14.57 ± 3.58	14.54 ± 3.87	6 months	Massage therapy + Schroth therapy	Schroth therapy
Liu et al. (2024a) (24)	From July 2021 to June 2023	36	44	12.73 ± 1.35	13.08 ± 1.39	6 months	Massage therapy + Schroth therapy	Schroth therapy
Li et al. (2021) (23)	From January 2018 to January 2021	30	30	13.32 ± 2.61	13.51 ± 2.21	6 months	Massage therapy + Schroth therapy	Schroth therapy
Wu (2024) (35)	From June 2023 to February 2024	31	30	13	13	3 months	Massage therapy + Schroth therapy	Schroth therapy
Xu and Huang (2022) (37)	From March 2020 to March 2021	30	30	12.37 ± 1.25	12.57 ± 1.31	8 weeks	Massage therapy + Schroth therapy	Schroth therapy
Lu et al. (2022) (41)	From September 2018 to December 2020	20	20	13.8 ± 2.1	13.7 ± 3.9	6 months	Massage therapy + Schroth therapy	Schroth therapy
Liu et al. (2024) (25)	From October 2021 to August 2023	44	44	14	14	4 weeks	Massage therapy + Schroth therapy	Schroth therapy
Xia et al. (2022) (36)	From January 2018 to June 2020	31	31	13.67 ± 2.92	13.82 ± 2.48	6 months	Massage therapy + Schroth therapy	Schroth therapy

### Quality assessment of the included studies

**Figure 2:**
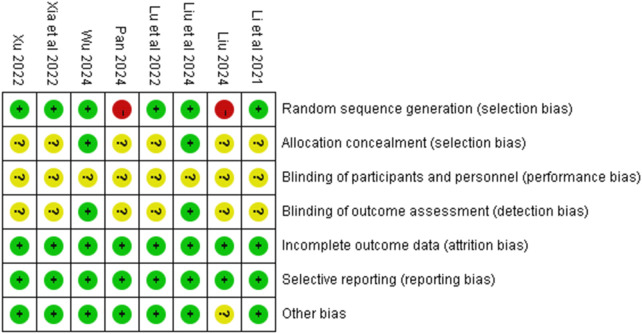
Risk of bias summary.

Among the included studies, 6 studies employed a random number table method for randomization and were rated as low risk of bias for sequence generation; the remaining two studies were rated as high risk. Two studies reported using an envelope method for allocation concealment, while the remaining studies provided insufficient information, resulting in unclear risk assessments for allocation concealment. Blinding of participants and personnel was generally unclear across studies. Two studies reported blinding of outcome assessors. All studies demonstrated low risk of bias regarding completeness of outcome data, selective reporting, and other sources of bias. Detailed risk of bias assessments are presented in Figure 2.

### Meta-analysis results

#### Cobb angle

**Figure 3:**
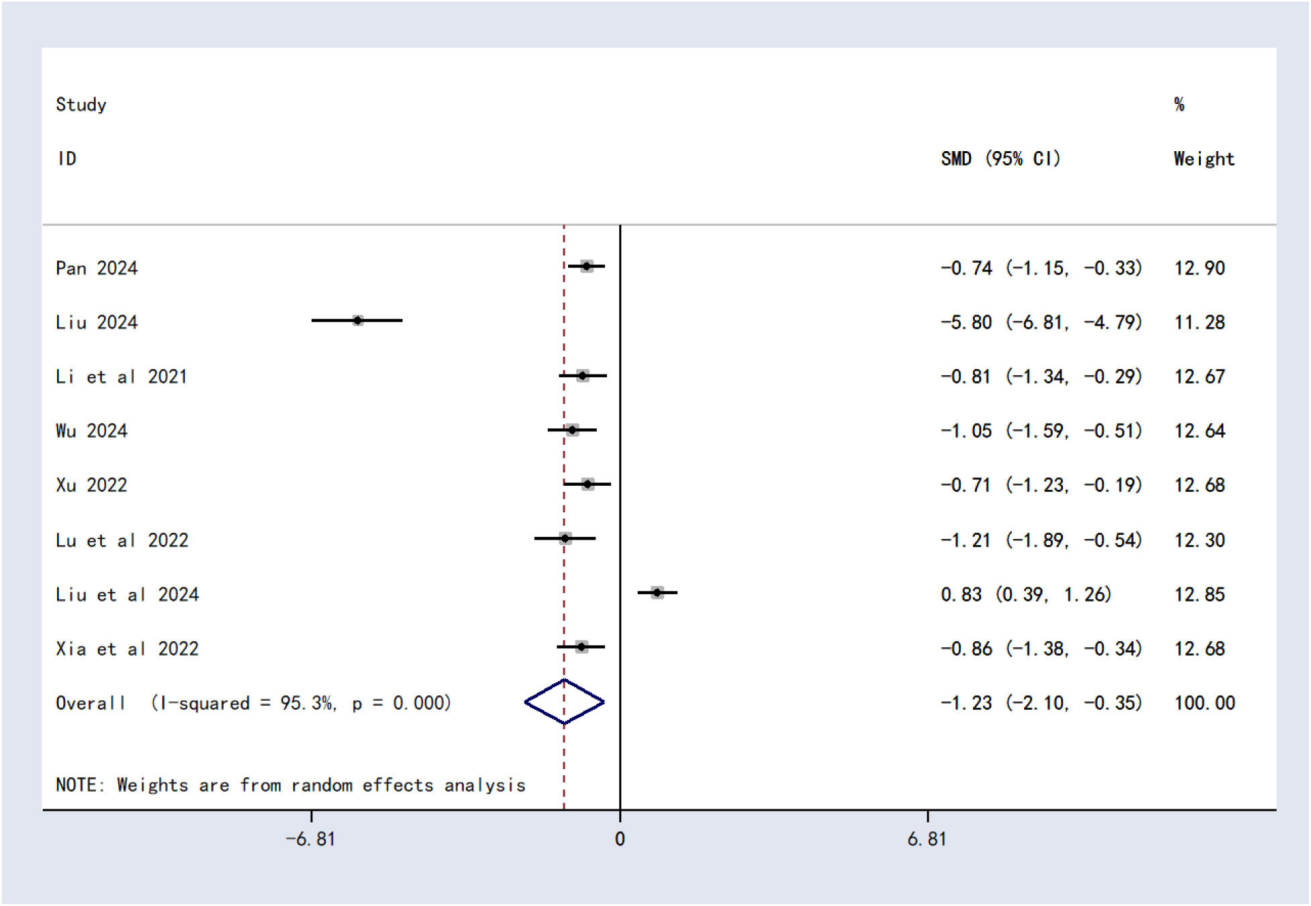
Forest plot of Cobb angle.

Eight studies involving 549 patients reported Cobb angle outcomes. Significant heterogeneity was detected (*I*^2^ = 95.3%, *P* < 0.00001), prompting the use of a random-effects model. Meta-analysis demonstrated that traditional Chinese massage therapy combined with Schroth therapy significantly reduced Cobb angle compared to Schroth therapy alone [SMD = −1.23, 95% CI (−2.10, −0.35), *P* = 0.006] (Figure 3).

#### Quality of life (SRS-22 score)

**Figure 4:**
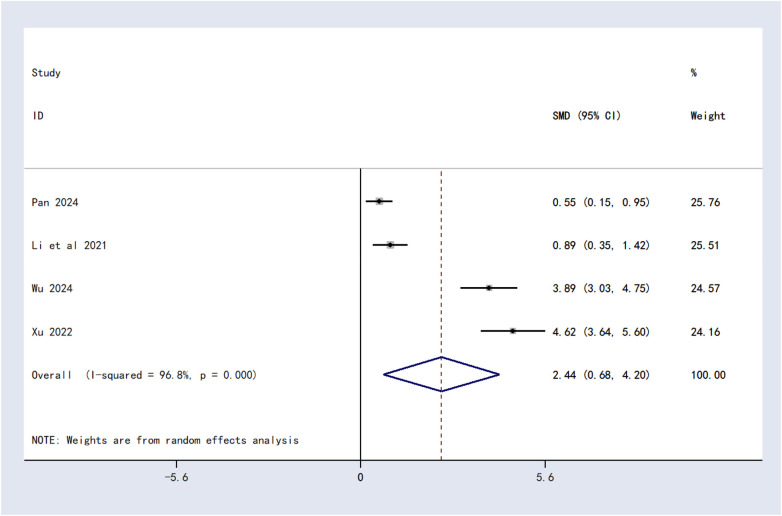
Forest plot of quality of life score.

Four studies involving 279 patients assessed SRS-22 scores. Considerable heterogeneity was observed (*I*^2^ = 96.8%, *P* < 0.00001), and a random-effects model was applied. The pooled results indicated that combined therapy significantly improved SRS-22 scores compared to Schroth therapy alone [SMD = 2.44, 95%CI (0.68, 4.20), *P* = 0.007] (Figure 4).

#### Total effective rate

**Figure 5:**
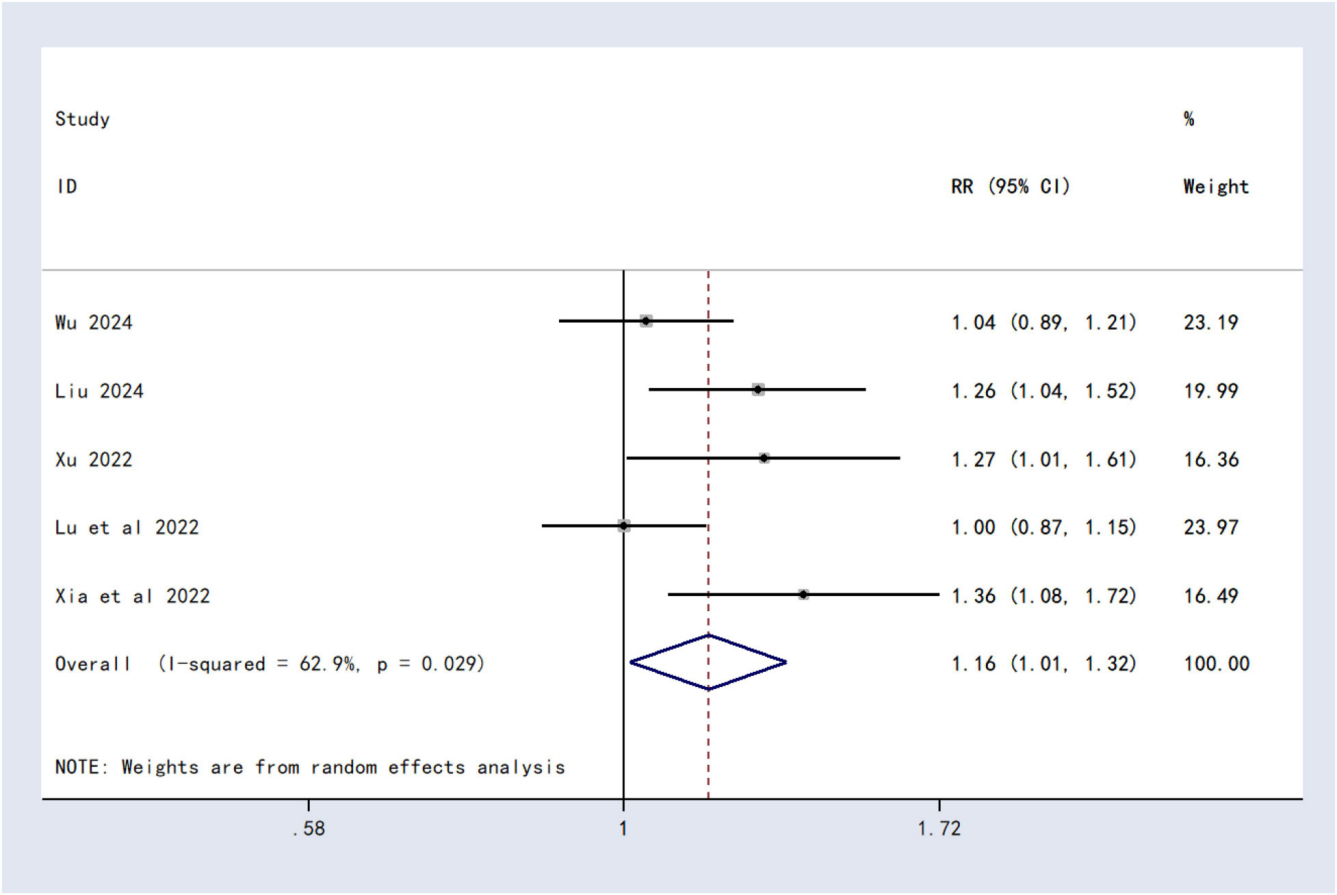
Forest plot of total effective rate.

Five studies involving 303 patients reported total effective rate. Moderate heterogeneity was detected (*I*^2^ = 62.9%, *P* = 0.029), and a random-effects model was employed. Meta-analysis showed that the combination therapy significantly improved total effective rate compared to Schroth therapy alone [RR = 1.16, 95% CI (1.01, 1.32), *P* = 0.0146] (Figure 5).

### Subgroup analysis

Subgroup analyses were conducted based on intervention duration (≥6 months vs. <6 months). The results are summarized in Table 2.

**Table 2:** Subgroup analysis.

Subgroups	*n*	Heterogeneity(I2)	RR/SMD(95%CI)	*P* value
Total effective rate	5	62.90%	RR = 1.16, 95%CI(1.01, 1.32)	*P* = 0.0146
≥6 months	3	76.70%	RR = 1.18, 95%CI(0.95, 1.48)	*P* = 0.137
<6 months	2	57%	RR = 1.13, 95%CI(0.92, 1.39)	*P* = 0.256
Cobb angles	8	95.30%	SMD = −1.23, 95%CI(−2.10, −0.35)	*P* = 0.006
≥6 months	5	95.40%	SMD = −1.81, 95%CI(−3.01, −0.6)	*P* = 0.003
<6 months	3	94.20%	SMD = −0.30, 95%CI(−1.49, 0.89)	*P* = 0.62
Scoliosis Research Society-22	4	96.80%	SMD = 2.44, 95%CI (0.68, 4.20)	*P* = 0.007
≥6 months	2	0.00%	SMD = 0.67, 95%CI (0.35, 1.00)	*P* < 0.00001
<6 months	2	16.70%	SMD = 4.21, 95%CI (3.56, 4.86)	*P* < 0.00001

CI, confidence interval; SMD, the standard mean difference; RR, relative risk.

For total effective rate, no statistically significant difference was observed between the two groups regardless of intervention duration (*P* > 0.05).

For Cobb angle reduction, combined therapy was significantly more effective when the intervention duration was ≥6 months (*P* < 0.05); however, no significant difference was found when the intervention duration was <6 months (*P* > 0.05).

For SRS-22 score improvement, combined therapy demonstrated significant benefits regardless of whether the intervention duration was ≥6 months or <6 months (*P* < 0.05).

### Sensitivity analysis

**Figure 6:**
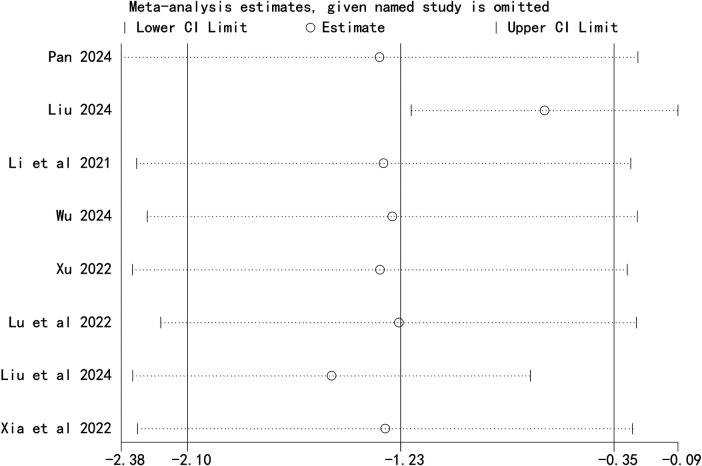
Sensitivity analysis of Cobb angle.

**Figure 7:**
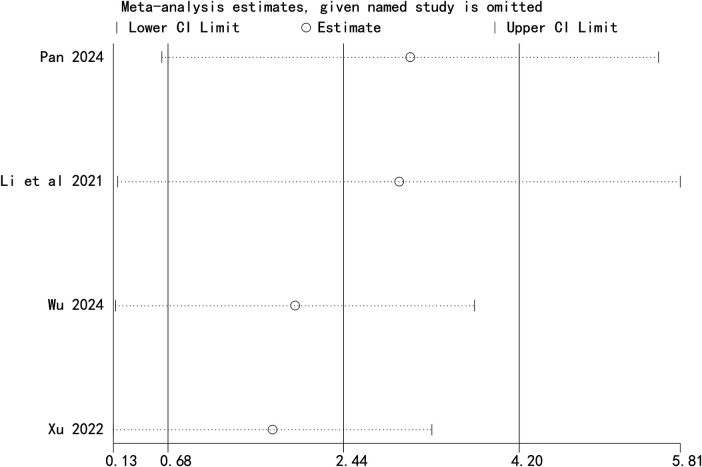
Sensitivity analysis of quality of life score.

**Figure 8:**
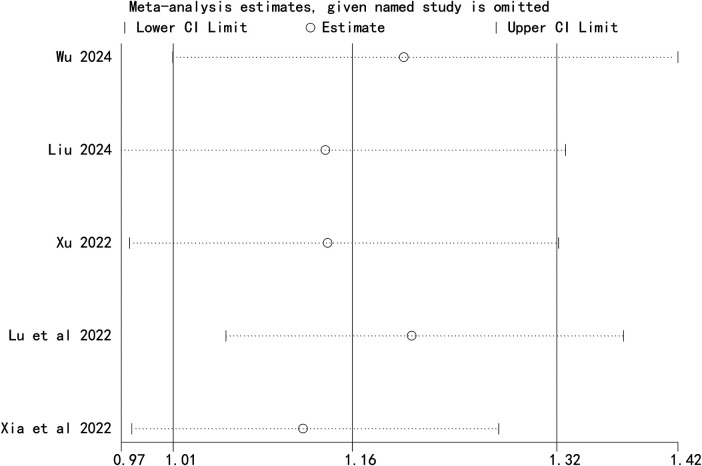
Sensitivity analysis of total effective rate.

Sensitivity analysis revealed that the pooled result for total effective rate was unstable, whereas the results for Cobb angle and SRS-22 score remained robust (Figures 6–8).

### Publication bias

**Figure 9:**
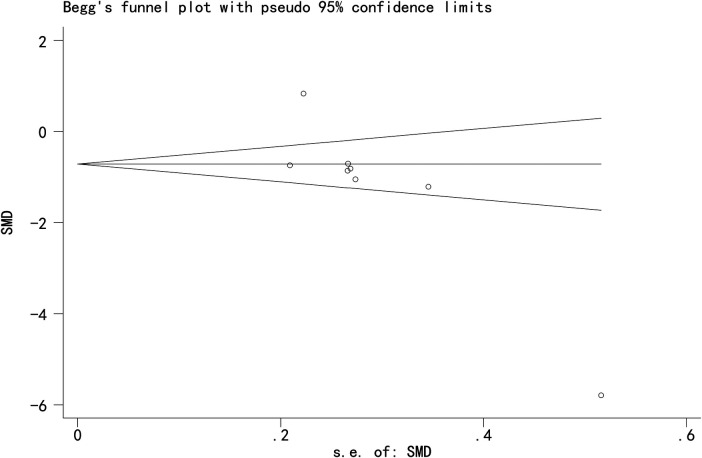
Funnel plot of Cobb angle.

**Figure 10:**
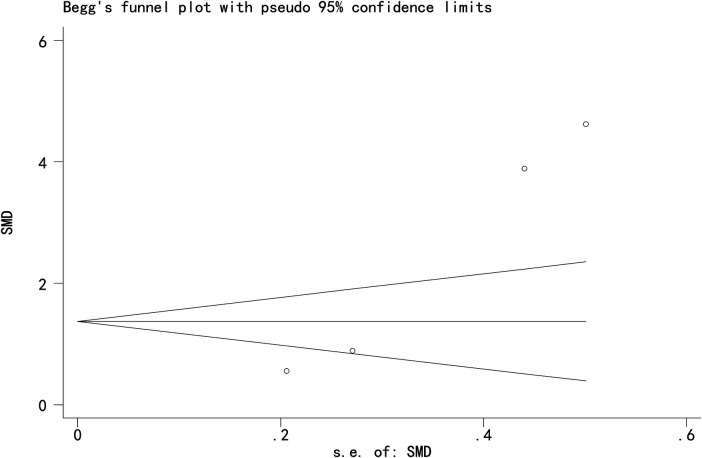
Funnel plot of quality of life score.

**Figure 11:**
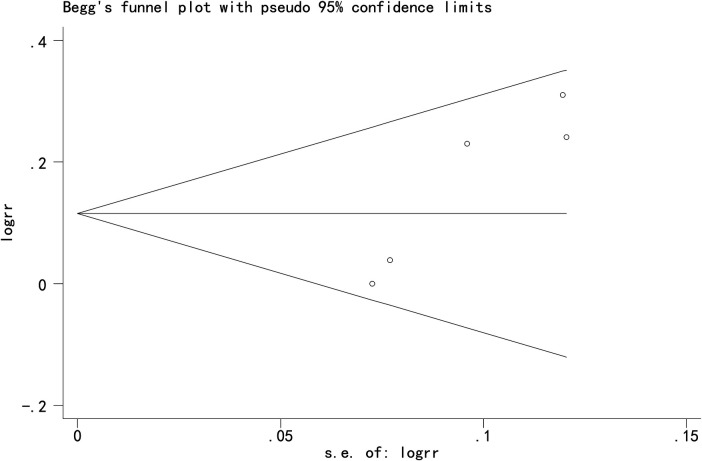
Funnel plot of total effective rate.

Egger's and Begg's tests indicated potential publication bias for the three primary outcomes: Cobb angle: Egger's test *P* = 0.009, Begg's test *P* = 0.013; SRS-22 score: Egger's test *P* = 0.017, Begg's test *P* = 0.042; Total effective rate: Egger's test *P* = 0.015, Begg's test *P* = 0.142 (Figures 9–11).

### Assessment of evidence quality

According to the GRADE methodology, the quality of evidence for all three outcomes was rated as very low (Table 3).

**Table 3:** GRADE systematic evaluation of evidence quality.

Outcomes	Quality assessment					Intervention/Control	RR/SMD(95%CI)	Certainty of the evidence
	Risk of bias	Inconsistency	Indirectness	Imprecision	Publication bias			
Total effective rate	Serious^a^	Serious^b^	Not serious	Serious^c^	Serious^d^	148/155 (5 studies)	RR = 1.16, 95%CI(1.01, 1.32)	Very low:⊕◯◯◯
Cobb angles	Serious^a^	Serious^b^	Not serious	Serious^c^	Serious^d^	271/278 (8 studies)	SMD = −1.23, 95%CI(−2.10, −0.35)	Very low:⊕◯◯◯
SRS-22	Serious^a^	Serious^b^	Not serious	Serious^c^	Serious^d^	140/139 (4 studies)	SMD = 2.44, 95%CI (0.68, 4.20)	Very low:⊕◯◯◯

a Some RCTs did not mention using the randomised scheme hiding or randomized grouping.

b Significant heterogeneity.

c The study sample size is small.

a Quantitative publication bias detection was statistically significant; CI, confidence interval; SMD, the standard mean difference; RR, relative risk; SRS-22, scoliosis research society-22.

## Discussion

This meta-analysis, encompassing eight randomized controlled trials involving 549 patients, demonstrates that compared to Schroth therapy alone, the combination with traditional Chinese massage therapy significantly reduces the Cobb angle and improves SRS-22 scores in patients with AIS. These findings hold considerable clinical relevance. For patients with mild to moderate AIS (Cobb angle <45°), a significant reduction in the Cobb angle may indicate effective deceleration of radiological curve progression, potentially reducing the future need for bracing or even surgical intervention. Concurrently, the observed improvement in SRS-22 scores directly reflects an enhancement in patients' quality of life. Therefore, this combined therapeutic approach not only offers a novel optimization strategy for the conservative management of AIS but also provides evidence-based support for clinical decision-making.

In traditional Chinese medicine, AIS falls within the categories of “hunchback” and “waist tilt” (42). Its etiology and pathogenesis are consistently attributed to underlying bodily deficiencies, including immature and weak muscles and bones, and deficiencies in qi, blood, and body fluids. Treatment strategies primarily target the spine and the Governor Vessel, and are considered closely related to the functional states of the liver, spleen, and kidney (43, 44). As a frequently employed external therapy in Chinese medicine, Tuina (traditional Chinese massage) utilizes various manipulative techniques, such as spinal manipulation and muscle kneading, to alleviate paravertebral muscle tension, dredge meridians, harmonize visceral functions, and regulate qi and blood circulation (35). Recent clinical studies have provided objective evidence supporting the use of Tuina for AIS. Tian et al. (45) randomized 90 AIS patients into two groups receiving either general Tuina or segmental spinal Tuina once weekly for 12 weeks. Their results indicated that segmental spinal Tuina effectively modulated the turns analysis of electromyographic signal recruitment in paravertebral muscles, improving the activation patterns of muscles on the convex and concave sides. The observation group demonstrated a significantly greater reduction in Cobb angle post-treatment compared to the control group (*P* < 0.05). This study offers electromyographic confirmation of Tuina's mechanism in improving spinal biomechanics. A more recent randomized controlled trial involving 116 AIS patients compared a control group receiving brace correction combined with postural training against an observation group receiving acupuncture combined with bone-setting Tuina. Results showed that the observation group exhibited significantly lower post-treatment Cobb angles and VAS pain scores (*P* < 0.01), along with significantly higher total effective rates and SRS-22 quality of life scores (*P* < 0.05) compared to the control group (46). Furthermore, Wang et al. (47), in a retrospective study of 262 AIS patients undergoing three-dimensional printed orthotic surgery, found that those receiving postoperative rehabilitation incorporating acupuncture and Tuina demonstrated significantly superior outcomes in trunk rotation angle, maximum Cobb angle, Oswestry Disability Index (ODI), and VAS pain scores compared to those receiving conventional rehabilitation alone (*P* < 0.05). Collectively, these studies indicate that traditional Chinese massage can effectively improve spinal function, alleviate pain, and enhance quality of life in AIS patients. Its advantages—including mild discomfort, minimal side effects, no impact on appearance, and favorable therapeutic outcomes—have contributed to its widespread acceptance among patients (37, 48, 49). Schroth therapy constitutes an exercise intervention specifically developed for distinct scoliotic deformities and curve types (50). Grounded in principles of muscle movement sensation and sensorimotor integration, it encompasses active and passive self-postural correction, repetitive exercises, and strength and endurance training of postural muscles. The therapy emphasizes conscious maintenance of correct posture during daily activities to mitigate muscle imbalance on either side of the spine by altering asymmetric body loading, alleviate back pain and sleep disturbances in scoliosis patients, and ultimately enhance self-image and quality of life (21, 28, 51). In recent years, multiple meta-analyses have provided robust evidence supporting the effectiveness of Schroth therapy. A network meta-analysis by Wang et al. (52) demonstrated that Schroth therapy was more effective in reducing Cobb angle compared to usual care. Notably, Schroth therapy also showed superior efficacy in Cobb angle reduction when compared to the Scientific Exercise Approach to Scoliosis (SEAS). Furthermore, Schroth therapy exhibited a significant advantage in improving quality of life scores. Previous meta-analyses have consistently shown that Schroth therapy significantly improves quality of life scores relative to control groups receiving usual care (52–55) Additional positive outcomes associated with Schroth therapy include enhancements in back muscle strength, respiratory function, pain reduction, and improvements in positive self-image (54–56).

Our subgroup analysis revealed that in the intervention duration ≥6 months subgroup, the addition of massage therapy effectively reduced Cobb angle. This observation aligns with existing literature suggesting that prolonged physical therapy can enhance spinal flexibility and muscle strength, thereby ameliorating structural spinal deformities (56). Moreover, massage therapy may potentiate the effects of Schroth therapy by promoting blood circulation and relaxing muscles, thereby engendering a synergistic effect. Conversely, in the <6 months intervention subgroup, massage therapy combined with Schroth therapy failed to demonstrate a statistically significant difference in Cobb angle improvement. This finding may be attributable to insufficient intervention duration, wherein short-term treatment may not fully activate the combined effects of both modalities, resulting in no appreciable clinical improvement. Consequently, for patients with scoliosis, extended intervention periods may constitute a critical determinant of achieving therapeutic goals. Notably, although intervention duration <6 months did not yield significant differences in Cobb angle improvement, massage therapy combined with Schroth therapy significantly enhanced SRS-22 scores irrespective of intervention duration. These findings suggest that while short-term interventions may have limited impact on structural spinal parameters, combined therapy can still exert positive effects on patients' quality of life and subjective perceptions. This phenomenon may be related to massage therapy's established roles in pain relief, muscle relaxation, and psychological state improvement. Future research should systematically investigate the influence of varying intervention durations on therapeutic outcomes in AIS patients and explore additional combined treatment strategies, ultimately aiming to provide more comprehensive treatment protocols for AIS.

### Limitation of this study

Although the results of this meta-analysis support the efficacy of traditional Chinese massage therapy combined with Schroth therapy for AIS, several important limitations warrant consideration. First, the number of included studies and their methodological quality were limited, resulting in the overall quality of evidence being rated as very low according to GRADE criteria. This substantially constrains the reliability and generalizability of our findings. Second, individual patient characteristics and variations in treatment protocol implementation may influence therapeutic efficacy, suggesting that personalized treatment approaches tailored to specific patient conditions are necessary in clinical practice. Third, this study is subject to potential publication bias and selection bias. Although systematic efforts were undertaken to minimize these biases through comprehensive literature searches, their potential influence on the results cannot be completely excluded. Consequently, the findings should be interpreted with appropriate caution. These limitations may restrict the global applicability of the study results. Future research should prioritize well-designed, multicenter randomized controlled trials with extended follow-up periods to validate these findings.

## Conclusion

In conclusion, the findings of this meta-analysis indicate that traditional Chinese massage therapy combined with Schroth therapy for AIS appears to yield superior outcomes in reducing Cobb angle, improving SRS-22 scores, and enhancing total effective rate compared to Schroth therapy alone. Notably, none of the included studies reported whether adverse events were monitored or occurred. Future studies should systematically monitor and report adverse events to enable comprehensive risk-benefit assessment.However, it must be emphasized that, owing to the limited quantity and quality of the included studies, the quality of evidence supporting these conclusions is rated as very low. In clinical practice, physicians may consider integrating traditional Chinese massage as a complementary option to Schroth therapy. Prior to implementing combined treatment, patients and their families should be fully informed regarding the limitations of the current evidence quality, and regular assessment of therapeutic efficacy and safety during the treatment course is recommended. The evidence quality rating of “very low” primarily stems from methodological deficiencies in the included studies—particularly concerning randomization concealment and blinding implementation—as well as potential publication bias. Therefore, while these findings contribute to guiding clinical practice, they also explicitly illuminate the fragility of the current evidence base. This recognition itself represents a significant contribution to the clinical research field: it delineates the direction for future investigation—namely, the urgent need for rigorously designed, adequately powered, and properly reported high-quality randomized controlled trials to further validate the precise efficacy of combined therapy and provide more reliable evidence-based guidance for AIS clinical management.

## Data Availability

The datasets presented in this study can be found in online repositories. The names of the repository/repositories and accession number(s) can be found in the article/Supplementary Material.
